# Clinical assessment of transthoracic echocardiography skills: a generalizability study

**DOI:** 10.1186/s12909-015-0294-5

**Published:** 2015-02-01

**Authors:** Dorte Guldbrand Nielsen, Signe Lichtenstein Jensen, Lotte O’Neill

**Affiliations:** 1 Department of Cardiology, Aarhus University Hospital, Aarhus, Denmark; 2 Center for Medical Education, Aarhus University, Aarhus, Denmark

**Keywords:** Transthoracic echocardiography, Echocardiography, Assessment, Ultrasound, Generalizability study, Decision study

## Abstract

**Background:**

Transthoracic echocardiography (TTE) is a widely used cardiac imaging technique that all cardiologists should be able to perform competently. Traditionally, TTE competence has been assessed by unstructured observation or in test situations separated from daily clinical practice. An instrument for assessment of clinical TTE technical proficiency including a global rating score and a checklist score has previously shown reliability and validity in a standardised setting. As clinical test situations typically have several sources of error giving rise to variance in scores, a more thorough examination of the generalizability of the assessment instrument is needed.

**Methods:**

Nine physicians performed a TTE scan on the same three patients. Then, two raters rated all 27 TTE scans using the TTE technical assessment instrument in a fully crossed, all random generalizability study. Estimated variance components were calculated for both the global rating and checklist scores. Finally, dependability (phi) coefficients were also calculated for both outcomes in a decision study.

**Results:**

For global rating scores, 66.6% of score variance can be ascribed to true differences in performance. For checklist scores this was 88.8%. The difference was primarily due to physician-rater interaction. Four random cases rated by one random rater resulted in a phi value of 0.81 for global ratings and two random cases rated by one random rater showed a phi value of 0.92 for checklist scores.

**Conclusions:**

Using the TTE checklist as opposed to the TTE global rating score had the effect of minimising the largest source of error variance in test scores. Two cases rated by one rater using the TTE checklist are sufficiently reliable for high stakes examinations. As global rating is less time consuming it could be considered performing four global rating assessments in addition to the checklist assessments to account for both reliability and content validity of the assessment.

**Electronic supplementary material:**

The online version of this article (doi:10.1186/s12909-015-0294-5) contains supplementary material, which is available to authorized users.

## Background

Transthoracic echocardiography (TTE) is a widely used cardiac imaging technique applied for the diagnosis and monitoring of numerous cardiac conditions. As a consequence, TTE is a procedure that all cardiologists should be able to perform competently [1-4]. In order to assure cardiology trainee competency, different methods of assessment have been suggested. Traditionally, competence assessment has been based on duration of training and a required minimum number of examinations performed [1-4]. However, in recent years TTE competence has also been evaluated by different national and international accreditation programs involving TTE technical proficiency and TTE knowledge in a high stakes examination [5]. Such high stakes examinations aim to assure sufficient technical proficiency and knowledge at a certain level of training, but do not necessarily provide information on actual daily clinical performance [6].

In a previous study, we described the development of an assessment instrument for TTE technical proficiency and explored the reliability and validity of the instrument in a standardised setting [7]. Under these controlled circumstances, the instrument showed evidence of validity based on positive correlations between test scores and competence level as well as evidence of reliability based on intraclass correlations (ICC) for both intra- and inter-rater reliability. ICC is a reliability index rooted in Classical Test Theory (CTT) which considers the observed test scores as consisting of two main components – a true score and error associated with the observation [8]. The ICC calculated in our previous study only takes one parameter, the observer, into account. However, since clinical test situations typically have more than one source of error, we need to know more about the impact of case and observer variance on the TTE assessment instrument scores in order to be able to generalise assessment scores to daily clinical competence [8].

According to modern validity theory, evidence of sufficient generalizability, or the degree to which we may generalize from observed scores to a universe score, is a form of construct validity evidence [9]. The Generalizability Theory (GT) as described by Brennan [10] is an extension of Classical Test Theory, which in contrast to CTT allows for the disentanglement of more than one source of error (e.g. rater and cases and occasion etc.) in a test situation. Since most real life test situations typically do have more than one source of error giving rise to variance in scores, reliability coefficients rooted in GT (‘generalizability’ coefficients) are more often than not less biased estimates of reliability, than the more commonly used CTT coefficients (e.g. Cronbachs’ alpha, Intraclass correlation coefficients and kappa coefficients etc.) [9,10]. The aim of this study was to thoroughly examine the reliability (generalizability) of the TTE technical proficiency instrument by means of Generalizability Theory. The objectives were to: 1) examine the concurrent influences of case- and observer effects on assessment scores, and 2) examine the optimal combination of numbers of cases and raters necessary to reach very high levels of reliability in test situations.

## Methods

### Material

A total of nine physicians participated in the study based on a sample size calculation [7]. Three novice echocardiographers (interns), three cardiology residents with some TTE experience, and three cardiology consultants with substantial echocardiography experience were recruited from the local university hospital and a local regional hospital. Novice echocardiographers were volunteer interns with no previous experience in TTE who received a total of four hours of TTE training prior to entering the study. The residents were in their first to third year of cardiology training and had some experience with TTE, but had not yet reached the level of a TTE expert. The consultants all worked with echocardiography in their daily practice and were considered experienced echocardiographers. As we made no further restrictions on the participants who could participate in this study, we believe that they are not systematically different from other physicians in our universe of admissible participants representing these three levels of competence. According to the Generelizability Theory the participants can therefore be considered a random facet [10]. Participation was voluntary and all participants signed a written consent. The study was presented to the local ethical review board, which did not find further approval necessary.

The nine physicians all performed a TTE scan of the same three patients. The three patients were randomly recruited in our outpatient clinic based on a desire to include a variety of significant and frequent pathologies and patients presenting different technical challenges in image acquisition. One patient was a younger male with a normal TTE scan and optical acoustic windows, another patient presented an aortic stenosis and had somewhat limited acoustic windows because of breast tissue, and the third patient was a male with a mitral regurgitation and challenging acoustic windows due to scar tissue from previous cardiac surgery. The physicians were asked to perform a full TTE scan of each patient based on Danish Cardiology Society (DCS) guidelines, which is a total of 26 images [11]. A list of the DCS recommendations was available to the physicians throughout the TTE scan.

Two raters rated all 27 TTE scans independently, that is the three TTE scans from all nine physicians. Both raters were cardiology consultants and clinical supervisors of cardiology trainees randomly invited from a larger pool of potential and equally admissible raters in our hospital. Rater 1 participated in the development of the assessment instrument, as he was involved in setting criteria for image rating as part of our previous study [7]. However, these predefined criteria for image grading was equally available for the second rater and hence both raters are considered equally capable of performing the ratings.

### Instrument

The assessment instrument consisted of a global rating scale and a procedure specific checklist. Common for both parts of the assessment instrument was a five-point scale ranging from (1) very poor (unsuitable for interpretation) to (5) very good (exceptionally good images). The global rating scale resulted in one score from 1 to 5 and providing an overall assessment of the quality of the TTE scan including number and quality of images as well as focus on relevant pathology. The procedure specific checklist on the other hand provided feedback on all requested images for as well anatomical presentation as optimization of screen window and technical settings. All relevant factors for the 26 requested images were rated using the checklist. A total checklist score of maximum 440 was calculated. A full description of the assessment instrument can be found in our previous work [7].

### Design

The study design was a fully crossed, all random generalizability study design of the form p x r x c [10]. In this ‘p crossed with c crossed with r’ design, the object of measurement (‘p’) is the physician, while ‘r’ represents raters, and ‘c’ the cases/patients scanned. The fully crossed design implies that all raters independently rated the same group of physicians on the exact same performances, i.e. the same TTE scans. When raters and cases are considered ‘random facets’ in generalizability terms it means, that the researchers did not by design put any restrictions on which raters or cases/patients from the larger universes of admissible raters and cases could be included in this study. The total observed variance in scores resulting from this test situation can be broken down into seven variance components (σ^2^) [10]:

1 $$ {\upsigma}_{obs}^2 = {\upsigma}_p^2 + {\upsigma}_r^2 + {\upsigma}_c^2 + {\upsigma}_{pr}^2 + {\upsigma}_{pc}^2+{\upsigma}_{rc}^2 + {\upsigma}_{prc,e}^2 $$

**Figure 1:**
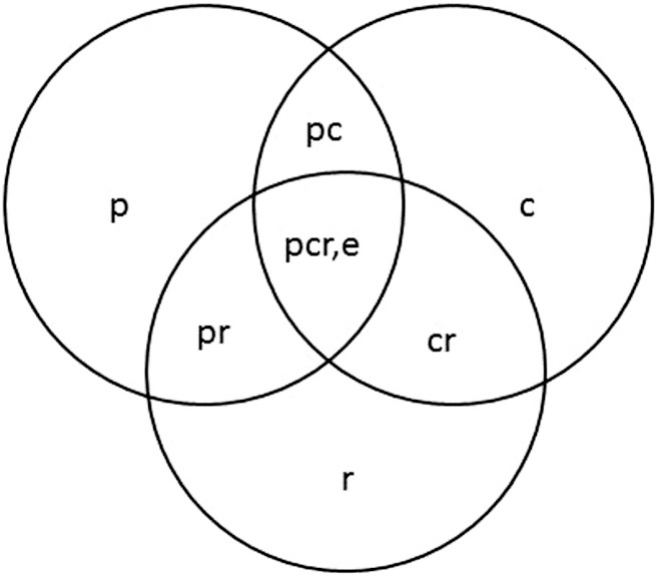
**Venn diagram of the variance components disentangled with the p x r x c design[**10**].** P = physician, r = rater, c = case, and e is any systematic error variance not disentangled as well as random error.

These seven variance components are explained individually in Table 1. The p, r and c components are main effects, whereas the rest are interaction effects. Figure 1 is a visual representation of all effects disentangled in this study.

**Table 1:** **Variance components for the p x r x c design explained**

Variance component	Explanation
σ^2^_p_	The variance in scores attributable to real differences in residents’ performances on TTE. This is known as the ‘universe score’ variance in GT. The equivalent in CTT is the ‘true score’ variance, σ^2^_τ_.
σ^2^_r_	The variance in scores attributable to rater differences in rating, e.g. differences in knowledge, skills and attitudes (e.g. ‘hawk’ or ‘dove’ attitudes) of raters.
σ^2^_c_	The variance in scores attributable to the case/patient. Some patients/conditions are easier to scan than others.
σ^2^_pr_	The variance in scores attributable to the interaction or ‘chemistry’ between physician scanning style and rater.
σ^2^_pc_	The variance in scores attributable to the interaction between residents and patients/cases. Different residents may perform differently based on the specifics of the case/patient.
σ^2^_rc_	The variance in scores attributable to the interaction between the rater and cases/patients. Different raters may rate differently based on the specifics of the case/patient.
σ^2^_prc,e_	The residual, which includes interaction between all effects (p, r and c) plus any systematic error variance not identified, as well as random error (e).

With estimates of the variance components in equation 1 from a generalizability (G) study, it is possible to calculate generalizability coefficients for any alternative test situation, i.e. for the use of alternative numbers of cases (n_c_) and numbers of raters (n_r_). Such calculations are called Decision (D) studies. This allows for determining which test situations are sufficiently reliable, and at the same time maximally feasible in the clinical setting. A generalizability coefficient for the absolute values of the TTE global rating scores and the total checklist scores respectively, for a number of alternative test situations may be calculated with Eq. 2, where raters and cases are considered random representatives of the universes of raters and cases [10].

2 $$ {\Phi}_{D(random)}=\frac{\upsigma_p^2}{\upsigma_p^2+\frac{\upsigma_r^2}{n_r}+\frac{\upsigma_c^2}{n_c}+\frac{\upsigma_{pr}^2}{n_r}+\frac{\upsigma_{pc}^2}{n_c}+\frac{\upsigma_{rc}^2}{n_r{n}_c}+\frac{\upsigma_{prc,e}^2}{n_r{n}_c}\ } $$

Phi (Φ), also known as the ‘Index of Dependability’ or the ‘dependability coefficient’, is the type of generalizability coefficient, which is appropriate in our case, as we are interested in absolute values of scores as opposed to ranks of scores. As can be seen, Eq. 2 is of the same basic form as the general equation used to calculate the reliability coefficients (R) rooted in Classical Test Theory (Eq. 3) [9].

3 $$ \mathrm{R} = \frac{\sigma_{\tau}^2}{\ {\sigma}_{\tau}^2 + {\sigma}_{\epsilon}^2} $$

where tau (τ) refers to true score and epsilon (ε) to error [9]. The only difference being, that in equation 2 multiple and specific sources of error variance is disentangled $\left({\upsigma}_r^2 + {\upsigma}_c^2 + {\upsigma}_{pr}^2 + {\upsigma}_{pc}^2+{\upsigma}_{rc}^2 + {\upsigma}_{prc,e}^2\right)$ instead of the single unspecific error variance component (σ^2^_ε_) in equation 3. As seen in Eq. 2, increased sampling of raters and cases (increases in n_c_ and n_r_) results in a decrease of the error variances (all variance components except σ^2^_p_) with a corresponding factor. So increased sampling of raters and cases increases test reliability or ‘dependability’ (Φ) as it is called here. Therefore the results of a G-study are useful when planning clinical test situations, i.e. where there is a need for test administrators to control reliability and optimize the use of resources (e.g. raters and cases in our case).

In circumstances where rater is considered a fixed facet, the test situation is better described by a mixed (random and fixed) generalizability design, and the phi-coefficient may be calculated using equation 4 [10].

4 $$ {\Phi}_{D(mixed)}=\frac{\upsigma_p^2+\frac{\upsigma_{pr}^2}{n_r}}{\upsigma_p^2+\frac{\upsigma_{pr}^2}{n_r}+\frac{\upsigma_c^2}{n_c}+\frac{\upsigma_{pc}^2}{n_c}+\frac{\upsigma_{rc}^2}{n_r{n}_c}+\frac{\upsigma_{prc,e}^2}{n_r{n}_c}\ } $$

Considering raters as fixed would be reasonable, if researchers deliberately excluded some raters from the larger universe of admissable raters as potential raters in the study *based on some particular characteristic*, so that the raters they ended up sampling were *systematically (not randomly) different* from the raters in the universe of admissable raters.

### Analysis

GENOVA for PC (Robert L Brennan, IowaTesting Programs, University of Iowa, Iowa City, IA, USA) was used to estimate the variance components in the G-study. GENOVA, which is freely available for download, uses Analysis of Variance (ANOVA) to estimate variance components [12]. Based on these estimates, we subsequently performed a series of decision studies (D-studies), in which dependability coefficients for test situations with different combinations of numbers of raters and cases were calculated with equation 2 by GENOVA, for both the TTE global rating score and the TTE total checklist scores respectively. We calculated dependability coefficients for test situations in which random raters are used (equation 2) and for a situation using a fixed rater pair (equation 4). A full output of the GENOVA studies for the global rating scores can be found in Additional file 1 and for the checklist scores in Additional file 2.

## Results

The generalizability-study results with the variance component value estimates are presented in Table 2 for TTE global rating and total checklist scores respectively. Using TTE global rating scores, only 66.6% of the total observed score variance can be ascribed to true differences in physician performance. In contrast, when relying on TTE total checklist score, true differences in physician performance accounted for as much as 88.8% of the total variance in scores (Table 2). As seen in Table 2, the error variances arising from the interaction effects in particular (pr, pc, rc and prc,e effects) accounted for an increasing proportion of the total variance in scores when global rating scores were used (31.5%), compared to when total checklist scores were used (6.9%). Of these error variances, the relative contribution of the physician-rater interaction effect in particular increased by a factor five when using global rating scores instead of total checklist scores (Table 2).

**Table 2:** **G-study results: estimated variance components with the p x c x r design for two types of scores**

	TTE global rating scores				TTE total checklist scores			
VC	Estimate	SE	% of total	d.f.	Estimate	SE	% of total	d.f.
P	0.949	0.487	66.6	8	11398.458	5220.439	88.8	8
R	0.000	0.016	0.0	1	339.363	313.301	2.6	1
C	0.028	0.050	1.9	2	215.851	187.863	1.7	2
pr	0.144	0.092	10.1	8	258.600	179.965	2.0	8
pc	0.102	0.069	7.1	16	232.329	157.676	1.8	16
rc	0.032	0.037	2.3	2	0.000	34.302	0.0	2
prc,e	0.171	0.057	12.0	16	394.993	131.664	3.1	16

TTE = transthoracic echocardiography, VC = variance components, SE = standard error, d.f. = degrees of freedom.

This situation generally resulted in lower dependability (phi) coefficients for TTE global rating scores than for TTE total checklist scores in comparable test situations (Table 3). As seen in Table 3, physicians would have to scan 2 different patients/cases with one random rater judging each situation using the TTE total checklist scores, for the test to reach dependability coefficients suitable for a high stakes test situation (Φ > 0.90) [13]. In contrast, if TTE global rating scores were used, the physicians would have to scan at least 4 cases, each to be rated independently by three random raters for comparable results (Table 3). As both raters and cases were a random sample of the universes of admissible raters and cases, the results may also be generalized to a test situation with any rater and patient/case within this universe. In contrast, if we restrict our universe of generalization to one containing only our two particular raters, i.e. if we consider rater as a fixed facet, even less sampling of patients/cases and raters is needed to reach dependability coefficients of 0.90 (D-study 2 in Table 3). In the following we will treat our results as all random, as we believe that our raters do represent a random sample of possible raters.

**Table 3:** **D-study results: dependability coefficients (Φ) in alternative test situations for two types of TTE scores with the p x r x c all random design (D-study 1) and with the p x r x c raters fixed design (D-study 2)**

	D-study 1						D-study 2			
	p x r x c, all random design (Eq. 2 )						p x r x c, raters fixed (Eq. 4 )			
n _cases_ \n _raters_	TTE global rating			TTE total checklist			TTE global rating		TTE total checklist	
	1	2	3	1	2	3	1	2	1	2
1	0.67	0.76	0.79	0.89	0.92	0.94	0.72	0.82	0.91	0.95
2	0.75	0.84	0.87	0.92	0.95	0.96	0.81	0.90	0.94	0.97
3	0.79	0.86	0.89	0.93	0.96	0.97	0.85	0.93	0.95	0.98
4	0.81	0.88	0.91	0.93	0.96	0.97	0.87	0.95	0.96	0.99
5	0.82	0.89	0.92	0.94	0.96	0.97	0.88	0.96	0.96	0.99

## Discussion

In this study we aimed to explore the impact of case- and observer variance on the assessment scores of a transthoracic echocardiography technical proficiency assessment instrument and examine how many raters needed to rate how many cases to establish sufficient reliability of the assessment score. The assessment instrument consisted of both a global rating score reflecting an overall rating of the TTE scan performed and a procedure specific total checklist score providing a more specific evaluation of each image performed.

The type of patient scanned as well as the rater simultaneously influenced the scores obtained by the physician in the test situation (Table 2). The reliability of scores also depended on whether the TTE global rating or the TTE checklist was used to assess physicians’ performances (Table 3). Using the TTE checklist as opposed to the TTE global rating score had the effect of minimising the three largest sources of error variance in test scores (Table 2). Most notably, the relative influence of the error variance attributable to the interaction or the ‘chemistry’ between rater and physician scanning style (the pr effect) was reduced with a factor 5 (from 10.1% to 2.0% of the total variance in scores). This means that biases such as ‘horn or halo’ effects [14] were more effectively curbed with the checklist than with global ratings. In addition, the relative influence of the error variance attributable to physicians of different competency levels interacting with patient cases of different difficulty (the pc effect) was reduced by a factor 3 using the checklist instead of global ratings (Table 2). This effect is often one of the most influential sources of error variance in educational assessments. Therefore, it has also been acknowledged for decades now in medical education, that as human performances are very much content or case specific, a principle known as the ‘content specificity’ of performances, it is of utmost importance to sample performance across a sufficient number of cases or patients or subjects for reliable performance scores [15]. Finally, the residual error variance (the prc,e effect) was reduced with a factor 4 when using the checklist instead of the global ratings.

We found that physicians would have to scan at least 2 different, random patients/cases with one randomly selected rater judging each situation using the TTE procedure specific checklist, for the assessment to be sufficiently reliable for a high stakes examination (Φ > 0.90). However, in order to avoid construct-underrepresentation and to improve content validity of the test [10], we consider it strongly advisable to include more than two cases in the assessment in spite of the high reliability scores for the TTE checklist. As global rating is less time consuming it could be considered performing four global rating assessments (Φ > 0.80) in addition to two checklist assessments to account for both reliability and content validity of the assessment.

The scientific method entails reproducible experiments and the use of reliable test instruments. This is equally true for test situations across most scientific disciplines whether they be medicine, psychology, medical education or other. Assessments in medical education must be reliable, so that outcomes or scores may be trusted, meaningfully interpreted, and resulting decisions defended [13]. Decisions based on assessment data in medical education may ultimately affect every day patient care as well as the progression of the trainee, and they are therefore not without consequences for stakeholders. In education multiple factors and in particular examinee competency level, rater stringency, item/case difficulty and the test occasion, are known to commonly influence test scores *simultaneously* [10,16,17]. Classical test theory (CTT) measures of reliability are usually not sufficiently suitable in such circumstances, because they only allow for accounting for one source of error at a time, i.e. either rater *or* case *or* occasion effects. Thus, an inter-rater reliability coefficient (ICC or kappa) which only accounts for a rater effect, or a coefficient alpha which only accounts for an item effect, or a test-retest reliability coefficient which only accounts for an occasion effect, are more often than not biased estimates of actual test situations in medical education [10]. Therefore, an increasing number of reliability studies in medical education use generalizability theory to estimate generalizability coefficients of workplace-based assessments, clinical performance assessments, admission tests etc. [17-33]. Besides offering the possibility of estimating less biased reliability coefficients in complex test situations, generalizability studies also invite researchers to calculate coefficients for alternative test situations based on the initial variance component analysis, via the second step in the process, which is the decision (D) study [10]. This may help in devising an optimal future test strategy with regards to both test feasibility and reliability simultaneously, as we have shown above (Table 3). This is often extremely useful, because in most test situations both in general education and in clinical training in hospital settings, there are not unlimited resources (e.g. raters, patients) available for testing purposes. The knowledge gained from a generalizability study offers an informed way out of unreliability, which is usually not apparent to researchers faced with the results of common CTT reliability measures (e.g. inter-rater ICC, kappa or a Cronbach’s alpha). In addition, generalizability studies require that researchers are completely aware of the match between test situation and type of coefficient used, and of any limitation in the universe of generalization. This helps researchers in avoiding an incorrect choice of coefficient and in over-interpreting results.

### Strength and limitations

The time of the day or the month, the order of examinations etc., i.e. the occasion may also affect human (physician, patient, rater) behaviour. The main limitation of this study is therefore, that we did not disentangle an occasion effect in addition to the rater and case effects examined. If we had, our G-coefficient could also have accounted for the test stability of the TTE technical proficiency assessment instrument. This would however have required a repetition of the whole test set-up on a second occasion, which was not feasible in the setting. It is likely that the occasion also affects test scores at least to some extent, and so the coefficients presented in this study cannot be said to be completely free of bias. In addition, there may be other systematic sources of error not sampled, which may also bias results such as the setting – an outpatient clinic or a ward. Still, the results represent less biased estimates of reliability than the inter-rater ICC coefficient on its own.

The limited sampling (in particular of raters) is a limitation of this study. With increased sampling of all facets (physicians, cases and raters) the relatively large SE of the estimated variance components seen in Table 2 could have been reduced, which would have strengthened the confidence in the results presented. However, we accepted the limitation of including only nine physicians performing three cases rated by two raters, as the task of rating 27 TTE scans requires a substantial time demand for a working clinician.

Another potential limitation is that one of the raters in the study participated in the development of the assessment instrument. However, this does not seem to be a significant limitation as the error variances arising from the variance between raters only account for 2.6% of total variance in the total checklist scores and has no influence on the total variance in global rating scores (Table 2).

It is a strength of this study, that we were able to examine a fully crossed G-design as this is the strongest type of GT design [10]. Also, another strength of the study is that the results provide us with information on how to best eliminate possible errors in daily clinical practice. That is, do we prefer more scans to be assessed or more raters to assess depending on whether we aim at high stakes examinations with a high reliability score or a formative assessments with lower reliability scores and a higher feasibility in daily clinical practice. However, the study does not provide us with information on how many cases to include assuring content validity of possible pathologies and technical difficulties.

## Conclusions

The results of this generalizability study indicate that the TTE technical proficiency assessment instrument can be feasibly applied to a clinical setting, as only two cases needed to be rated by one randomly available rater for each examinee in order to reach very high levels of reliability.

## Addititional files

Additional file 1: **Presents the full output data of the GENOVA analysis of the global rating scores.** Data for both all random and mixed analysis with raters as a fixed facet is included. Additional file 2: **Presents the full output data of the GENOVA analysis of the checklist scores.** Data for both all random and mixed analysis with raters as a fixed facet is included.

## Abbreviations

TTE: Transthoracic echocardiography
ICC: Intraclass correlations
CTT: Classical Test Theory
GT: Generalizability Theory
DCS: Danish Society of Cardiology

## References

[1] Quinones MA, Douglas PS, Foster E, Gorcsan J, Lewis JF, Pearlamn AS (2003). ACC/AHA Clinical Competence Statement on Echocardiography: a report of the American College of Cardiology/American Heart Association/American College of Physicians-American Society of Internal Medicine task force on clinical competence. J Am Coll Cardiol.

[2] Ryan T, Armstron WF, Khandheria BK (2008). Task Force 4: Training in Echocardiography. J Am Coll Cardiol.

[3] Popescu BA, Andrade MJ, Badano L, Fox KF, Flachskampf F, Lancellotti P (2009). European Association of Echocardiography recommendations for training, competence, and quality improvement in echocardiography. Eur J Echocardiogr.

[4] Bauersachs J, Bax J, Burri H, Caforio ALP, Calvo F, Charron P (2013). ESC Core Curriculum for the General Cardiologist. Eur Soc Cardiol Committ Educ Eur Heart J.

[5] Fox KF, Popescu BA, Janiszewski S, Nihoyannopoulos P, Fraser AG, Pinto FJ (2007). Report on the European Association of Echocardiography Accreditations in Echocardiography: December 2003 - September 2006. Eur J Echocardiogr.

[6] Rethans J-J, Norcini JJ, Barón-Maldonado M, Blacmore D, Jolly BC, LaDuca T (2002). The relationship between competence and performance: implications for assessing practice performance. Med Educ.

[7] Nielsen DG, Gotzsche O, Eika B (2013). Objective structured assessment of technical competence in transthoracic echocardiography: a validity study in a standardized setting. BMC Med Educ.

[8] Streiner DL, Norman GR (2003). Health Measurement Scales.

[9] Kane MT, Brennan RL (2006). Validation. Educational Measurement.

[10] Brennan RL (2001). Generalizability Theory.

[11] DCS (2008). Anbefalinger for standardiseret minimumskrav for transthorakal ekkokardiografi hos voksne. Recommendations for standardized minimum demands for adult transthoracic echocardiography.

[12] Genova programs, Center for Advanced Studies in Measurement and Assessment (CASMA); University of Iowa http://www.uiowa.edu/~casma/computer_programs.htm [Accessed 01 April 2014].

[13] Downing SM (2004). Reliability: on the reproducibility of assessment data. Med Educ.

[14] Thorndike EL (1920). A constant error in psychological ratings. J Appl Psychol.

[15] Norman G, Bordage G, Page G, Keane D (2006). How specific is case specificity?. Med Educ.

[16] Crossley J, Russel J, Jolly B, Ricketts C, Roberts C, Schuwirth L (2007). ‘I’m picking up good regressions’: the governance of generalizability analyses. Med Educ.

[17] Shavelson RJ, Webb NM (1991). Generalizability Theory. A primer.

[18] Sebok SS, Luu K, Klinger DA (2014). Psychometric properties of the multiple mini-interview used for medical admissions: findings from generalizability and Rasch analyses. Adv Health Sci Educ Theory Pract.

[19] Homer M, Setna Z, Jha V, Higham J, Roberts T, Boursicot K (2013). Estimating and comparing the reliability of a suite of workplace-based assessments: an obstetrics and gynaecology setting. Med Teach.

[20] Baig LA, Violato C (2012). Temporal stability of objective structured clinical exams: a longitudinal study employing item response theory. BMC Med Educ.

[21] Hanson MD, Kulasegaram KM, Woods NN, Fechtig L, Anderson G (2012). Modified personal interviews: resurrecting reliable personal interviews for admissions?. Acad Med.

[22] Lohfeld L, Goldie J, Schwartz L, Eva K, Cotton P, Morrison J (2012). Testing the validity of a scenario-based questionnaire to assess the ethical sensitivity of undergraduate medical students. Med Teach.

[23] Richter Lagha RA, Boscardin CK, May W, Fung CC (2012). A comparison of two standard-setting approaches in high-stakes clinical performance assessment using generalizability theory. Acad Med.

[24] Dornan T, Muijtjens A, Graham J, Scherpbier A, Boshuizen H (2012). Manchester Clinical Placement Index (MCPI). Conditions for medical students’ learning in hospital and community placements. Adv Health Sci Educ Theory Pract.

[25] Alves de Lima A, Conde D, Costabel J, Corso J, Van der Vleuten C (2013). A laboratory study on the reliability estimations of the mini-CEX. Adv Health Sci Educ Theory Pract.

[26] Karabilgin OS, Vatansever K, Caliskan SA, Durak Hİ (2012). Assessing medical student competency in communication in the pre-clinical phase: objective structured video exam and SP exam. Patient Educ Couns.

[27] Uijtdehaage S, Doyle L, Parker N (2011). Enhancing the reliability of the multiple mini-interview for selecting prospective health care leaders. Acad Med.

[28] Bergus GR, Woodhead JC, Kreiter CD (2010). Using systematically observed clinical encounters (SOCEs) to assess medical students’ skills in clinical settings. Adv Med Educ Pract.

[29] Weller JM, Jolly B, Misur MP, Merry AF, Jones A, Crossley JG (2009). Mini-clinical evaluation exercise in anaesthesia training. Br J Anaesth.

[30] Wass V, Jones R, Van der Vleuten C (2001). Standardized or real patients to test clinical competence? The long case revisited. Med Educ.

[31] Burch VC, Norman GR, Schmidt HG, van der Vleuten CP (2008). Are specialist certification examinations a reliable measure of physician competence?. Adv Health Sci Educ Theory Pract.

[32] Fernandez SA, Wiet GJ, Butler NN, Welling B, Jarjoura D (2008). Reliability of surgical skills scores in otolaryngology residents: analysis using generalizability theory. Eval Health Prof.

[33] Wass V, McGibbon D, Van der Vleuten C (2001). Composite undergraduate clinical examinations: how should the components be combined to maximize reliability?. Med Educ.

